# Editorial: Targeting immune cell metabolism in tissue fibrosis

**DOI:** 10.3389/fphar.2024.1400858

**Published:** 2024-04-05

**Authors:** Dan Shan, Qianjiang Hu, Chao He, Yong Zhou

**Affiliations:** ^1^ Section of Pulmonary Diseases, Critical Care and Environmental Medicine, John W. Deming Department of Medicine, Tulane University, New Orleans, LA, United States; ^2^ Division of Pulmonary, Allergy, Critical Care, and Sleep Medicine, Department of Medicine, University of Pittsburgh, Pittsburgh, PA, United States; ^3^ Division of Pulmonary, Allergy, and Critical Care Medicine, Department of Medicine, University of Alabama at Birmingham, Birmingham, AL, United States

**Keywords:** immune cell, fibrosis, metabolism, mitochondria, macrophage

Tissue fibrosis, characterized by the excessive accumulation of extracellular matrix contents, leads to chronic dysfunction across various organs, including the liver, lungs, heart, and kidneys. This pathological process disrupts normal tissue architecture and function, contributing to significant morbidity and mortality worldwide. Recent advances in our understanding of fibrosis have highlighted the pivotal role of immune cell metabolism in fibrogenesis. Immune cells, by modulating their metabolism, can either promote or attenuate the fibrotic process, providing novel perspectives through which we can understand and potentially treat fibrotic diseases. In this Research Topic, four seminal papers contribute to this evolving landscape, marking a commendable advancement in our understanding of fibrosis and related immunometabolic processes, and collectively pushing the boundaries of therapeutic development.

The study by Chang et al. provides a comprehensive analysis of Vitamin E’s impact on pulmonary fibrosis, revealing its multifaceted role in mitigating the disease progression. Through detailed experiments, including a bleomycin-induced murine lung fibrosis model, the research delineates the efficacy of Vitamin E in suppressing key fibrotic processes such as epithelial cell apoptosis, fibroblast activation, epithelial-mesenchymal transition, and tissue inflammation. Moreover, it underscores a distinct process by which orally administered Vitamin E maintains iron homeostasis and mitochondrial function *in vivo*, thereby attenuating fibrosis. Interestingly, this function acts independently of the established antioxidant effects of Vitamin E. The study identifies Vitamin E as a promising agent in mitigating disease development and progression, offering a novel therapeutic pathway for treating pulmonary fibrosis.

The study conducted by Xu et al. examined the effects of total glucosides of paeony (TGP) on intestinal immune imbalance and epithelial barrier damage in rats with collagen-induced arthritis (CIA). The investigators demonstrate that TGP regulates inflammatory responses by alleviating immune cell hyperactivation and enhancing epithelial barrier integrity after collagen exposure. Utilizing metabolomics and network pharmacology analyses, the paper elucidates the complex mechanisms by which TGP exerts its protective effects on the gut mucosa. This work not only underscores the importance of gut immunity in systemic autoimmune diseases but also advances our understanding of the multifaceted therapeutic actions of TGP, highlighting its potential as a treatment option for autoimmune diseases, through the modulation of gut immunity and restoration of epithelial functions.

The review article by Feng et al., “Immunometabolism changes in fibrosis: from mechanisms to therapeutic strategies,” offers a comprehensive summary of the immunometabolism in fibrotic diseases. This review meticulously delineates the latest research on metabolic reprogramming in immune cells and fibroblasts, aiming to bridge gaps between metabolic dysregulation and fibrotic disease progression. Through a detailed analysis of altered metabolic pathways, this review enriches our understanding of fibrosis pathophysiology and highlights the potential of targeting metabolic reprogramming to combat fibrotic diseases.

The review article by Zhang et al., titled “Research Progress on Mesenchymal Stem Cells and Their Exosomes in Systemic Sclerosis,” offers a detailed examination of the potential of mesenchymal stem cells (MSCs) and MSC-derived exosomes as innovative therapies for systemic sclerosis (SSc). The authors thoroughly explore the mechanisms through which MSCs and their exosomes can address key pathological processes in SSc, such as interstitial lung disease, skin fibrosis, and endothelial proliferation. By summarizing current research findings, the paper offers unique insights into the therapeutic potential of MSCs and exosomes, illuminating a promising therapeutic strategy that leverages the regenerative capabilities of stem cells to address the complexities of SSc.

In summary, the four papers in this Research Topic collectively highlight recent advances in understanding how immune cells contribute to the development of fibrosis and highlight the promise of targeting immune cell metabolism as a strategy for treating fibrotic conditions. From harnessing the therapeutic potential of natural compounds and vitamins to exploring the cutting-edge realm of stem cell therapy, these studies broaden the horizons of fibrosis treatment. As the field continues to evolve, it is clear that continued research into the metabolic underpinnings of fibrosis holds great promise for developing innovative, effective, and targeted treatments. The evolution of fibrosis treatment may well hinge on our ability to harness the power of metabolic modulation, marking a new era in our approach to combating this pervasive and challenging disease.

